# Seasonal Patterns in Erectile Dysfunction Severity

**DOI:** 10.7759/cureus.91295

**Published:** 2025-08-30

**Authors:** Hector R Gonzalez-Carranza, Luis A Reyes-Vallejo

**Affiliations:** 1 Department of Urology, Hospital Angeles Metropolitano, Mexico City, MEX

**Keywords:** associated factors, epidemiology and public health, erectile dysfunction, erection hardness score, global epidemiology, iief-5 score, male sexual dysfunction, male sexual health, public health, risk factors

## Abstract

Background

Erectile dysfunction (ED) is a prevalent condition affecting millions of men worldwide. Risk factors for ED are known to be more prevalent during some seasons, yet the potential seasonal variation in ED symptom severity remains underexplored. This study investigates the relationship between the time of year and the clinical intensity of ED symptoms using the International Index of Erectile Function (IIEF-5).

Methodology

Between August 2023 and March 2025, 202 patients presenting with ED were assessed and classified into severity groups according to the IIEF-5 score. The Spearman’s correlation test was applied to assess the relationship between IIEF-5 scores and the month of symptom onset.

Results

Questionnaires from 202 patients were collected. The average age of the sample was 51 years, ranging from 22 to 82 years. Overall, 52 (25.7%) patients belonged to the mild severity group, 81 (40%) to the mild-to-moderate group, 50 (24.8%) to the moderate group, and 13 (6.4%) to the severe ED group. When categorized by months, the results were as follows: January 80 (39.6%), February 26 (12.87%), March 13 (6.44%), April 4 (1.98%), May 9 (4.46%), June 15 (7.43%), July 7 (3.47%), August 17 (8.42%), September 4 (1.98%), October 17 (8.42%), November 4 (1.98%), and December 6 (2.97%). Spearman’s test showed a correlation coefficient (ρ) of -0.15. The p-value was 0.03, indicating statistical significance at the 0.05 level.

Conclusions

This study demonstrates a statistically significant seasonal variation in the severity of ED symptoms, with more severe cases tending to present during the early months of the year.

## Introduction

Erectile dysfunction (ED), characterized by the persistent inability to attain or maintain an erection sufficient for satisfactory sexual performance, presents a multifaceted clinical challenge with varying degrees of severity [1]. ED severity is commonly assessed by the International Index of Erectile Function (IIEF-5). This validated questionnaire assesses patients’ confidence to achieve and keep an erection, the hardness to achieve penetration, the ability to maintain an erection after penetration and how difficult it is, and how often sexual relationships are satisfactory for the patient. This tool allows physicians to classify patients into the following groups: mild, mild-to-moderate, moderate, and severe [2].

By 2025, ED is projected to affect approximately 322 million men all over the world [3]. Several risk factors have been identified, yet the most important one is hypertension, followed by diabetes, lower urinary tract symptoms, depressive symptoms, and age [4]. These risk factors are known to be more prevalent during some seasons, particularly during winter. Blood pressure changes in cold weather, as the blood vessels clamp down trying to retain the heat [5]. Diabetes seasonal patterns have also been observed, with the highest rate found in fall and winter [6]. Similarly, winter has also been associated with worsening of depressive symptoms [7].

The seasonal variability of ED itself is still not well understood, despite these seasonal trends in known ED factors. Few studies have directly examined how the severity of ED varies with the seasons to date, which is a major gap in the literature and a crucial topic for further study.

Gul et al. performed a retrospective, observational study during 2021 and found that ED admissions are associated with higher peaks in the winter seasons [8]. This adds to the study performed by Garijo et al. in 2022, which showed an increase in searches for ED during winter. Both studies highlight the need for awareness of ED-associated lifestyle risk factors and aid patients in receiving proper and on-time diagnosis and treatments [9]. This study aims to identify the relationship between months of the year and the severity of ED.

## Materials and methods

A cross-sectional study was conducted between August 2023 and March 2025 at Hospital Ángeles Metropolitano, a tertiary care center located in Mexico City. The study aimed to evaluate the seasonal variation in ED severity. Because this was an observational, non-interventional study involving anonymous data collection without patient identifiers, Institutional Review Board approval was not required under local research ethics guidelines.

Inclusion criteria were adult male patients (≥18 years old) who presented for urological consultation with ED symptoms as their primary complaint and agreed to participate. Exclusion criteria included patients with incomplete questionnaires, those unable to recall the approximate onset month of their ED symptoms, and individuals with known psychiatric or neurological conditions affecting sexual function.

Patients were recruited consecutively during routine outpatient consultations using a non-probability convenience sampling technique. After providing verbal informed consent, eligible participants were asked to complete the IIEF-5 questionnaire. With strong test-retest reliability (r = 0.82-0.88) and high internal consistency (Cronbach’s α >0.90), the IIEF-5 is a validated and trustworthy instrument for evaluating erectile dysfunction. Higher scores indicate better erectile function. There are five questions on the test, with each one scored from 1 to 5. The total score ranges from 5 to 25. Patients were classified as mild (17-21), mild-to-moderate (12-16), moderate (8-11), or severe (5-7) ED based on predetermined cutoffs [10]. (license ID for questionnaire use number: 1638424-1). Additionally, they were asked to report the calendar month when they first noticed ED symptoms.

To explore the presence of a seasonal pattern in ED severity, the Spearman’s correlation test was applied to assess the relationship between the IIEF-5 scores and the month of symptom onset.

## Results

Questionnaires from 202 patients were collected. The average age of the study sample was 51 years, with a range from 22 to 82 years. Patients were categorized into severity groups according to their IIEF-5 results and cross-referenced with the month in which their symptoms started.

Based on the IIEF-5 questionnaire, we classified patients into mild, mild-to-moderate, moderate, and severe ED groups. We found that 52 (25.7%) patients belonged to the mild severity group, 81 (40%) to the mild-to-moderate group, 50 (24.8%) to the moderate group, and 13 (6.4%) to the severe ED group.

**Figure 1:**
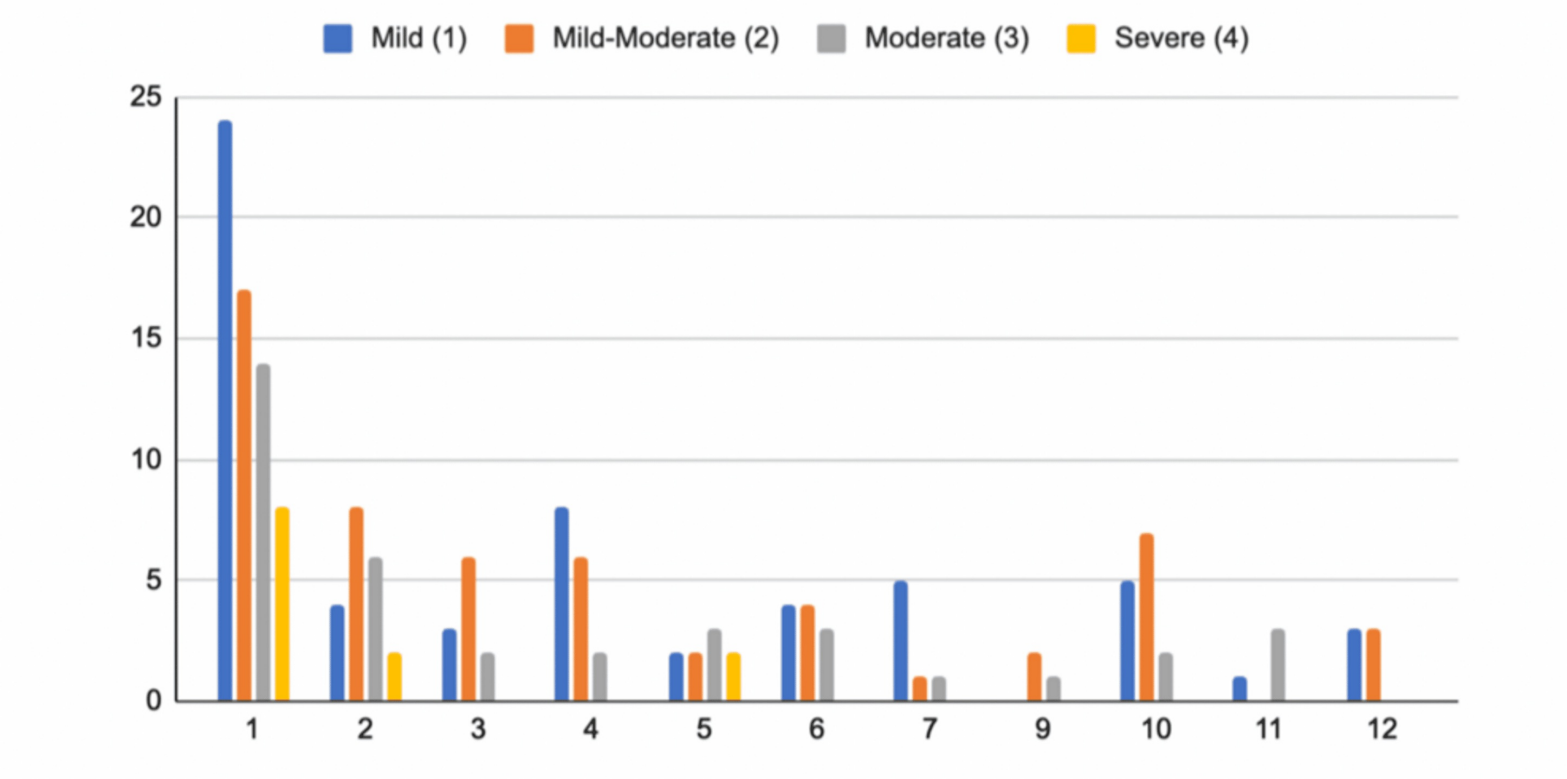
Erectile dysfunction severity by month of symptom onset. The x-axis represents the month of the year, and the y-axis represents the International Index of Erectile Function (IIEF-5) score.

When categorized by months, the results were as follows: January 80 (39.6%), February 26 (12.87%), March 13 (6.44%), April 4 (1.98%), May 9 (4.46%), June 15 (7.43%), July 7 (3.47%), August 17 (8.42%), September 4 (1.98%), October 17 (8.42%), November 4 (1.98%), and December 6 (2.97%) (Figure 1).

January was the month with the most patients reporting symptoms onset. Nine patients presented with severe ED, representing 13 (70%) of all the patients with severe ED across the study sample. February was the second month with the most patients, with two reporting severe ED; nevertheless, May had the same number of patients, while only presenting a total of nine patients. Months with the lowest incidence were April, September, and November. Of these patients, one (25%), two (50%), and three (75%), respectively, presented moderate ED.

Spearman’s test was used to establish a correlation between IIEF-5 severity results and months of the year. The correlation coefficient (ρ) was found to be -0.15. The p-value was 0.03, indicating statistical significance at the 0.05 level. A weak negative correlation suggested that as the number of months increases, the severity tends to slightly decrease, but the relationship was not strong. As the p-value was <0.05, we reject the null hypothesis, indicating that there is a statistically significant monotonic relationship between ED severity and the months of the year.

## Discussion

ED is a condition with multifactorial causes, including vascular, neurological, hormonal, and psychological components. While much attention has been directed toward these mechanisms, the influence of environmental or seasonal factors on the severity of ED symptoms has been largely underexplored [11,12]. This study contributes to the limited body of research investigating seasonality in ED, finding a statistically significant, albeit weak, negative correlation between the month of symptom onset and ED severity. In interpreting these findings, several key mechanisms and clinical implications warrant consideration.

One likely explanation for the observed seasonal pattern relates to lifestyle and behavioral factors that tend to fluctuate across the year. The colder months, particularly winter, are often associated with decreased physical activity, poorer dietary habits, and increased weight gain, each of which has direct implications for endothelial function, testosterone levels, and overall metabolic health [13,14]. As the pathophysiology of ED is closely linked to vascular integrity and hormonal balance, it is plausible that lifestyle regression during winter months might aggravate underlying erectile issues or trigger more severe manifestations in men who are already at risk [7,15].

Psychological health is also a central component in the experience and progression of ED, and it shows well-documented seasonal variation. Winter is associated with increased prevalence of depressive symptoms and seasonal affective disorder, conditions that not only impair libido and sexual function but also influence how men perceive and report the severity of their symptoms [16]. A man experiencing mild organic dysfunction in the context of seasonal depression may interpret his symptoms as more severe, or he may be less responsive to compensatory mechanisms that would otherwise maintain function. This could explain, in part, the clustering of more severe ED presentations during the early part of the year [17,18].

Hormonal rhythms may also contribute to this seasonal pattern. Several studies have reported cyclical fluctuations in testosterone levels, with lower concentrations during winter and higher levels in late spring or summer [19]. Given the central role of testosterone in sexual desire and erectile physiology, these hormonal shifts could plausibly modulate the clinical expression of ED [20]. While the magnitude of seasonal variation in testosterone is modest and likely insufficient to cause ED on its own, it may interact with other risk factors, such as stress, comorbid disease, and medication use, to produce a cumulative effect that pushes certain individuals into more severe clinical categories during specific times of the year.

Importantly, the findings of this study align with prior investigations that identified increased ED-related healthcare utilization and online search behavior during winter. The retrospective study by Gul et al. found higher rates of ED-related admissions during colder months, while Garijo et al. documented a winter peak in ED-related Google searches in the United States [12].

However, these studies did not measure the clinical intensity of the condition, focusing instead on health-seeking behavior. By directly examining ED severity, as classified by IIEF-5 scores, the current analysis adds a clinically relevant layer to the conversation. It suggests that seasonal variation may influence not only when men seek help but also how severely their symptoms are experienced at the time of onset.

These results support a broader model of ED as a dynamic condition, responsive not only to biological and relational variables but also to contextual and environmental factors. The implications of this perspective are significant for clinical care. Awareness of seasonal variation may help clinicians anticipate symptom fluctuations and adjust their screening or treatment strategies accordingly. For example, patients presenting in the winter with newly severe ED might benefit from early evaluation for cardiovascular risk factors or depressive symptoms, both of which may contribute to or be exacerbated by seasonal changes. Integrating questions about the timing of symptom onset into the clinical history could enhance diagnostic accuracy and guide personalized management strategies.

Furthermore, this seasonal awareness could be useful in planning public health messaging or educational campaigns. Knowing that men are more likely to experience or report severe ED during winter months allows for targeted outreach during periods of heightened vulnerability.

This study found a modest correlation, but it is consistent with the multifactorial nature of ED. ED, like many chronic conditions, is the result of the accumulation and interaction of multiple risk domains rather than a single trigger. In this context, even a weak but consistent seasonal influence may serve as a tipping point for symptom emergence or intensification. Similar studies in other areas of men’s health, such as mental illness and cardiovascular disease, which both exhibit higher morbidity and severity during the winter, lend credence to this theory.

Despite these insights, several limitations of the present study should be acknowledged. Recall bias may arise from the use of retrospective self-reporting of symptom onset. Patients may find it challenging to precisely date symptoms that appear gradually or intermittently, especially if they put off getting help. The study’s clinic-based sample may also limit generalizability, as individuals who seek specialized care for ED may differ in severity, socioeconomic status, or health awareness from those who manage symptoms in primary care or not at all. Additionally, the geographic and climatic characteristics of the study population, drawn from an urban setting in Mexico, may not fully capture the variability present in regions with more extreme seasonal changes.

Clinical trials could also be designed to test interventions tailored to seasonal risk periods. Likewise, investigating whether testosterone replacement or other hormonal interventions have greater efficacy or necessity during winter could refine treatment protocols. These approaches would move the field beyond documentation of seasonal patterns toward actionable strategies that improve patient outcomes.

In the context of current clinical guidelines, which largely treat ED as a temporally stable condition, this study challenges a static approach and encourages a more dynamic, time-sensitive perspective. While the month of symptom onset may seem a subtle detail, its association with symptom severity, however modest, suggests it could serve as a useful clinical cue. This is particularly relevant in settings where healthcare resources are limited, and a nuanced understanding of symptom patterns can enhance decision-making and patient prioritization.

## Conclusions

This study demonstrates a statistically significant seasonal variation in the severity of ED symptoms, with more severe cases tending to present during the early months of the year. These findings align with existing research on healthcare-seeking behavior and add novel insight by focusing specifically on clinical severity. The results suggest that environmental, psychological, and hormonal factors linked to seasonal changes may influence not only when men seek care for ED but also how intensely they experience their symptoms. As ED continues to be a sentinel marker of men’s health and quality of life, recognizing the temporal dimension of symptom variation may enhance clinical care, inform public health efforts, and guide future research. Ultimately, integrating seasonality into the understanding of ED can lead to more personalized and effective strategies for diagnosis, prevention, and treatment.
